# Alkaline Phosphatase Kinetics Predict Metastasis among Prostate Cancer Patients Who Experience Relapse following Radical Prostatectomy

**DOI:** 10.1155/2018/4727089

**Published:** 2018-06-28

**Authors:** Carolyn A. Salter, Jennifer Cullen, Claire Kuo, Yongmei Chen, Lauren Hurwitz, Adam R. Metwalli, Jordan Dimitrakoff, Inger L. Rosner

**Affiliations:** ^1^Department of Urology, Walter Reed National Military Medical Center, Bethesda, MD, USA; ^2^Center for Prostate Cancer Disease Research, Rockville, MD, USA; ^3^Department of Surgery, Uniformed Services University of the Health Sciences, Bethesda, MD, USA; ^4^Urologic Oncology Branch, National Cancer Institute, National Institute of Health, Bethesda, MD, USA; ^5^US Food and Drug Administration Division of Bone, Reproductive and Urologic Products, Silver Spring, MD, USA

## Abstract

**Introduction:**

Metastasis prostate cancer (CaP) occurs in a small fraction of patients. Improved prognostication of disease progression is a critical challenge. This study examined alkaline phosphatase velocity (APV) in predicting distant metastasis-free survival (DMFS).

**Materials and Methods:**

This retrospective cohort study examined CaP patients enrolled in the Center for Prostate Disease Research (CPDR) multicenter national database who underwent RP and experienced BCR (n=1783). BCR was defined as a PSA ≥ 0.2 ng/mL at ≥ 8 weeks post-RP, followed by at least one confirmatory PSA ≥ 0.2 ng/mL or initiation of salvage therapy. APV was computed as the slope of the linear regression line of all alkaline phosphatase (AP) values after BCR and prior to distant metastasis. APV values in the uppermost quartile were defined as “rapid” and compared to the lower three quartiles combined (“slower”). Unadjusted Kaplan Meier (KM) estimation curves and multivariable Cox proportional hazards analysis were used to examine predictors of DMFS.

**Results:**

Of the 1783 eligible patients who experienced post-RP BCR, 701 (39.3%) had necessary AP data for APV calculation. PSA doubling time (PSADT) and APV were strongly associated (p=0.008). No differences in APV were observed across race. In KM analysis, significantly poorer DMFS was observed among the rapid versus slower APV group (Log-rank p=0.003). In multivariable analysis, a rapid APV was predictive of a twofold increased probability of DMFS (HR = 2.2; 95% CI = 1.2, 3.9; p = 0.008), controlling for key study covariates.

**Conclusions:**

Building on previous work, this study found that rapid APV was a strong predictor of DMFS for a broader group of CaP patients, those who undergo post-RP BCR who were enrolled in a longitudinal cohort with long-term follow-up and equal health care access. APV is worth considering as a complementary clinical factor for predicting DMFS.

## 1. Introduction

Prostate cancer (CaP) is the most common nonskin cancer in the United States and the third leading cause of cancer-related death in men with 161,360 new cases and 26,730 deaths estimated in 2017[1]. The majority of men with prostate cancer do not die of their disease [1–3]. Development of distant metastasis is a useful surrogate of prostate cancer specific death. Clinical predictors of CaP progression have included PSA doubling time (PSADT) and more recently alkaline phosphatase (AP) velocity (APV) [4–6]. AP is a known marker of bone-turnover, specifically osteoblast activity. In prior studies, AP has been shown to be elevated in men with bony metastasis and AP elevation is correlated to metastatic burden [7]

Previous, related research conducted at the Center for Prostate Disease Research (CPDR) evaluated men with castrate-resistant CaP (CRPC) [4, 8]. Alkaline phosphatase velocity (APV) has also been studied as a means to predict progression in men with CaP. A recent article evaluating the CPDR multicenter national database examined men who had castrate-resistance prostate cancer (CRPC) following androgen deprivation therapy and reported that faster APV was a strong predictor in combination with PSA doubling time (PSADT) in predicting development of distant bone metastasis. A similar study retrospectively evaluated patients enrolled at Memorial Sloan Kettering Cancer Center to confirm findings from the CPDR study. In the MSKCC study, Hammerich et* al*. showed that, among patients with post-RP CRPC who received ADT for an elevated PSA, those who had rapid APV progressed more quickly to bone metastasis and had poorer overall survival compared to those with slower APV [9]

The primary study aim was to examine APV as a predictor of distant metastasis-free survival (DMFS) among men who experienced biochemical recurrence following radical prostatectomy (RP) in the context of an equal access health care system. A secondary aim was to confirm the joint roles of APV with PSADT in predicting DMFS.

## 2. Methodology

### 2.1. Study Design and Source of Study Subjects

The study population was comprised of men enrolled in the Institutional Review Board-approved Center for Prostate Disease Research (CPDR) multicenter national database (described in detail previously) [10]. Briefly, patients under suspicion for CaP are eligible for enrolment at five US military and 1 civilian medical center nationwide. A total of 8,041 men with biopsy-confirmed CaP detected between 1989 and 2013 and who underwent RP as primary treatment (i.e., within 6 months of CaP diagnosis) were eligible. The main exclusion criterion was no evidence of BCR during study interval (n=6,101). Patients were also excluded if they had less that 1-year follow-up (n=60) or if there was M1 disease at presentation (n=15) or positive nodal status at diagnosis (n=82).

### 2.2. Demographic, Clinical, Pathologic, and Treatment Information

For each subject, data were obtained on age at CaP diagnosis, age at RP, self-reported race, PSA at diagnosis (ng/mL), D'Amico risk stratum (low, intermediate, and high), pathologic T stage (T2, T3-4), pathologic grade (≤6, 3+4, 4+3, and 8-10), surgical margin status (positive, negative), extracapsular extension or ECE (positive, negative), and seminal vesicle invasion or SVI (positive, negative). PSA doubling time (PSADT) was calculated as previously described by Pound et al. [6] using all available PSA values at least 3 months apart after BCR. If the slope of the linear regression line was 0 (i.e., elevated but constant PSA levels) or negative (decreasing PSA levels after an initial increase), the PSADT was set to 10 years (120 months). Detailed information on all treatments received before were obtained and categorized as neoadjuvant pre-RP), adjuvant (post RP but pre-BCR), and salvage (post-BCR).

### 2.3. Assessment of Alkaline Phosphatase (AP) Kinetics

APV was computed as the slope of the linear regression line of all alkaline phosphatase (AP) values after RP and prior to METs. APV values in the uppermost quartile were defined as “rapid” and compared to those in the lower 3 quartiles combined. Finally, a kinetics measure of alkaline phosphatase velocity (APV) was calculated by using the slope of the linear regression line of the AP values plotted against time in years. This was computed using all AP values drawn at least 3 months apart and obtained after CRPC developed but before radiographic scan-detected metastasis to bone or other locations (e.g., visceral metastasis). APV was dichotomized at the observed upper quartile of all observed AP values in this study sample (<3.11 versus ≥3.11 U/L-year).

### 2.4. Primary Study Endpoint

The primary study endpoint was distant metastasis-free survival (DMFS). Presence of metastases was ascertained based on complete radiographic scan history (bone scan, CT scan, and MRI), captured as part of ongoing data collection activities for the CPDR multicenter national database. Time to DMFS was calculated as the number of years elapsed from time point of documented BCR to distant metastasis or until end of study period for those who did not experience distant metastasis.

### 2.5. Statistical Analysis

Descriptive statistics included means and standard deviations (SD), frequencies, and percentages. Student's* t*-tests or Wilcoxon-Mann-Whitney tests were used to compare distributions in continuous patient characteristics, including age and time variables, across APV groups. Mantel Haenzsel chi-square tests were used to examine differences in the distributions of categorical variables across APV groups.

Unadjusted Kaplan Meier (KM) estimation curves were used to model time to distant-metastasis-free survival (DMFS) across APV strata and PSADT strata. The log rank test and its associated p-value are reported for KM analyses. Multivariable Cox proportional hazards analyses were used to model DMFS controlling for demographic, clinical, pathologic, treatment, and time covariates. Hazard ratios (HR) and corresponding 95% confidence intervals (CI) and p-values are reported.

All statistical tests are 2-sided (summary alpha error = 0.05), and the decision rule was based on a p-value < 0.05. All statistical analyses were performed using SAS version 9.3 and R.

## 3. Results

**Figure 1:**
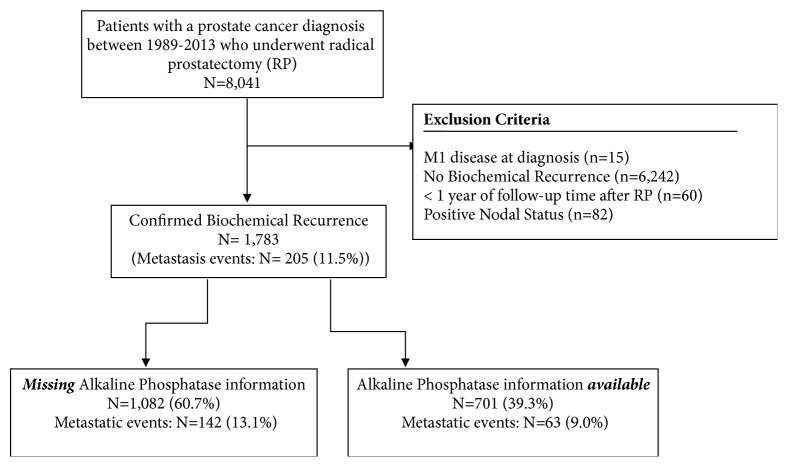
Flow diagram of study population selection.

Of the 1,783 patients who experienced post-RP BCR, sufficient alkaline phosphatase (AP) data were available for 701 (39.3%) subjects. This subset represents the study cohort (Figure 1). There were 63 patients (9%) who developed distant metastases during a mean follow-up time of 10.1 years. Among these 63 metastatic patients, 36 (57.4) were confirmed by bone scan, 24 (38.1%) were confirmed by CT scan, 2 (3.2%) were confirmed by MRI, and 1 patient (1.6%) was detected with a metastasis of CaP to the bladder. Per definition of the APV categorized variable, those with a “rapid” APV accounted for 25% of the cohort and the remaining 75% had a slower APV (Table 1). A majority of patients (62%) received some form of salvage treatment. Chi-square analysis did show a significant association between APV and PSADT categories (p=0.15).

**Figure 2:**
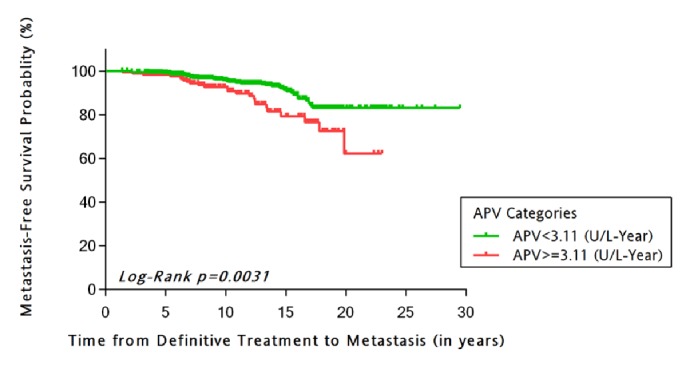
Unadjusted Kaplan-Meier estimation curve of distant metastasis-free probability stratified by alkaline phosphatase velocity (APV) categories.

**Figure 3:**
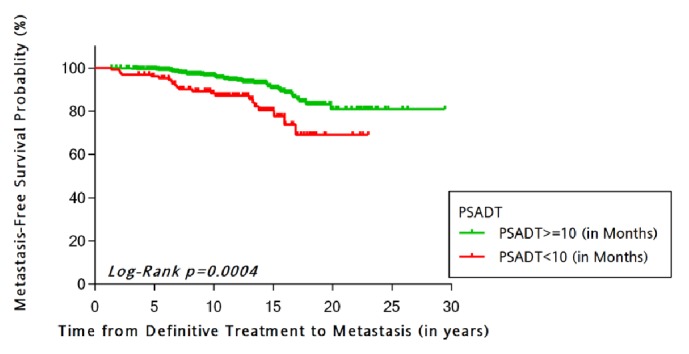
Unadjusted Kaplan-Meier estimation curve of distant metastasis-free probability stratified by PSA doubling time (PSADT) categories.

Unadjusted Kaplan-Meier estimation curves for DMFS stratified for APV (Figure 2) and PSADT (Figure 3) demonstrated that those with rapid versus slower APV group had poorer DMFS (p=0.003) as did patients with faster versus slower PSADT (<10 versus ≥10 months) (p=0.0004). KM analysis was also performed for PSADT cutpoints by Freedland et al. which demonstrated strong associated with DMFS-free survival (p<0.0001). Though there were few observations in the PSADT <3 months category, data are presented for Pound et* al*. cutpoints.

In multivariable Cox proportional hazards analysis, only rapid APV (HR_>=3.11  vs.  <3.11_ = 2.2; 95% CI=1.2, 3.9; p=0.008), PSADT (HR_<10  vs.  >=10_ = 2.0; CI=1.1, 3.8; p=0.03), and pathological Gleason (HR_8-10  versus  6_ =2.3; CI=1.0, 5.1; p=0.04) were statistically significant predictors of DMFS (Table 2).

Due to concerns over study bias that might have been caused by missing AP data, preventing calculation of APV, comparisons were made across the subsets of subjects with versus without adequate AP data, across demographic, clinical, pathologic, and treatment variables (Table 3). Among statistically significant differences noted, those* with* versus* without* AP data had a longer follow-up (10.1 versus 7.3 years, p<0.001), a greater proportion of salvage treatment (62% versus 50%, p<0.0001); a greater proportion of pT2 disease (50% versus 54%, p=0.01), and a lower proportion of ECE (38% versus 47%, p<0.0001).

## 4. Discussion

While routine examination of alkaline phosphatase (AP) in prostate cancer care was previously observed, the introduction and widespread use of PSA screening in the late 1980s greatly diminished its clinical use. This study, along with previous and related work, has shown that faster AP kinetics are predictive of poorer metastasis-free survival in distinct subsets of prostate cancer patients.

More recently there has been a renewed interest in using AP to predict disease progression. One study attempted to predict overall survival in men with metastatic CRPC and found that AP was predictive of survival (p= 0.027) whereas PSA was not (p=0.742) [11]. A similar paper which again used subjects with metastatic CRPC found a median AP of 172. When comparing men with AP ≤ 172 their median survival was 18 months compared to 10 months in men with AP > 172 (p< 0.001) [12]. These data show that, in men with metastatic CRPC, AP levels can be used to predict survival. Metwalli et* al*. looked at a similar question and found that, in men with CRPC, faster APV can predict progression to bone metastases. [9]

The current study focused on a time interval in the continuum of CaP treatment that is further upstream than previously examined, by analyzing the role of APV in predicting DMFS in patients who experience post-RP BCR. These data suggests that APV can be used to predict disease progression in numerous stages along trajectory of CaP care, from BCR, to CRPC to metastatic CRPC.

Strengths of this study include the longitudinal, racially diverse patient cohort with long-term patient follow-up in an equal-access health care system. Even though many of these patients were excluded due to lack of alkaline phosphatase data, we still have a large patient cohort to examine the significance of alkaline phosphatase velocity.

Limitations include the retrospective design, precluding the ability to examined temporal changes in short intervals of time on APV and outcome, DMFS. In addition, the role of neoadjuvant, adjuvant, and salvage therapies was limited to categories of any use versus none. Details on duration of use and medication type were beyond the scope of this study. Also, APV was categorized due to nonnormality and strong skew in its data distribution. But identifying an optimal cutpoint that would be useful in other cohorts is a challenge. In this study, unbiased categories were data-driven, with patients dichotomized into rapid versus slower groups, representing the uppermost quartile versus the lower 3 combined, respectively. This approach is limited and may not be externally generalizable.

## 5. Conclusions

This study builds on our previous work that demonstrated APV as a strong predictor of distant metastasis-free survival in men with CRPC. This study expanded the study question to a broader group of CaP patients—those who experience post-RP BCR—and demonstrated that APV is a strong predictor of DMFS in this patient subset as well. By examining patients at a time point upstream of CRPC, namely, time of BCR, this study provides support for APV as a tool for improved prognostication of DMFS. Future work should be extended to examine this question among patients undergoing radiation, with or without hormone therapy, who experience biochemical relapse.

## Figures and Tables

**Table 1:** Descriptive characteristics of study sample (n=701).

	**Study Cohort (N=701)**
**Continuous Variables**	**Mean ± SD**

**Age at CaP Diagnosis (years), Mean ± SD**	62.6 ± 6.4
**Age at RP (years), Mean ± SD**	62.8 ± 6.4
**Time from RP to Distant Metastasis (years), Mean ± SD**	10.1 ± 4.7

**Categorical Variables**	**N (**%**)**

**Distant Metastasis, N (**%**)**	63 (9.0)
**Neo- and/or Adjuvant Treatments Received, N (**%**)**	127 (18.1)
**Salvage Treatments Received, N (**%**)**	437 (62.3)
**PSA Doubling Time (Months), N (**%**)**	
Missing	22 (3.1)
<10	129 (18.4)
≥ 10	550 (78.5)
**D'Amico risk stratum, N (**%**)**	
Missing	165 (23.5)
Low	171 (24.4)
Intermediate	199 (28.4)
High	166 (23.7)
**Race, N (**%**)**	
Missing	8 (1.1)
African American	163 (23.3)
Caucasian	494 (70.5)
Other	36 (5.1)
**PSA at CaP Diagnosis, N (**%**)**	
Missing	126 (18.0)
<10	371 (52.9)
10-20	139 (19.8)
>20	65 (9.3)
**Pathologic Gleason Sum, N (**%**)**	
Missing	98 (14.0)
≤6	234 (33.3)
3+4	201 (28.7)
4+3	73 (10.4)
≥ 8	95 (13.6)
**Pathological T stage, N (**%**)**	
Missing	15 (2.1)
T2	334 (47.6)
T3-T4	352 (50.3)
**Positive Surgical Margin Status, N (**%**)**	311 (44.4)
**Positive Extracapsular Extension, N (**%**)**	265 (37.8)
**Positive Seminal Vesicle Invasion, N (**%**)**	96 (13.7)

**Table 2:** Multivariable Cox proportional hazards analysis of distant metastasis-free survival.

**Variable**	**Hazard Ratio (95% CI)**	**p-value**
**Age at RP** ^**1**^(years)	0.99 (0.95, 1.04)	0.78

**Time from RP to BCR** ^**2**^(years)	0.92 (0.81, 1.04)	0.18

**Neo- and/or Adjuvant Treatments Received**		

Yes vs. No	1.36 (0.69, 2.69)	0.38

**Post-BCR** ^**2**^ ** Salvage Treatment Received**		

HT Only vs. None	0.94 (0.45, 1.98)	0.87

XRT Only vs. None	0.47 (0.15, 1.41)	0.17

Multi-Treatment^*∗*^ vs. None	1.66 (0.73, 3.79)	0.23

**APV** ^**3 **^ **Upper Quartiles (U/L-Y)**		

≥3.11 vs. <3.11	2.18 (1.23, 3.86)	**0.008**

**PSADT** ^**4**^(Months)		

<10 vs. ≥10	2.01 (1.07, 3.78)	**0.030**

**Race**		

African American vs. Caucasian American & Other	1.07 (0.54, 2.13)	0.84

**Pathologic T stage**		

pT3-T4 vs. pT2	0.99 (0.47, 2.07)	0.97

**Pathologic Gleason Sum**		

3+4 vs. ≤6	1.1 (0.53, 2.28)	0.81

4+3 vs. ≤6	0.91 (0.32, 2.58)	0.85

≥8 vs. ≤6	2.29 (1.03, 5.07)	**0.042**

**Surgical Margin Status**		

Positive vs. Negative	1.08 (0.53, 2.19)	0.84

^1^RP=radical prostatectomy.

^2^BCR=biochemical recurrence.

^3^APV=alkaline phosphatase velocity.

^4^PSADT=PSA doubling time.

^*∗*^Multitreatment refers to combinations of XRT and HT.

**Table 3:** Comparison of patients with versus without alkaline phosphatase (AP) data needed for calculating AP velocity (APV).

	**Alkaline Phosphatase Data Available**		**p-value**
	*No* *(n=1,082)*	*Yes* *(n=701)*	
**Age at CaP** ^1^ ** Diagnosis **(years), Mean ± SD^2^	62.1 ± 7	62.6 ± 6.4	0.16
**Age at RP** ^**3**^(years), Mean ± SD	62.3 ± 7	62.8 ± 6.4	0.17
**Time from RP** ^3^ ** to Distant Metastasis **(years), Mean ± SD	7.3 ± 5.4	10.1 ± 4.7	**<.0001**
**Distant Metastasis, N (**%**)**	142 (13.1)	63 (9)	
**Neo- and/or Adjuvant Treatments Received, N (**%**)**	195 (18)	127 (18.1)	0.96
**Salvage Treatments Received, N (**%**)**	544 (50.3)	437 (62.3)	**<.0001**
**PSA Doubling Time (Months), N (**%**)**			0.36
Missing	69 (6.4)	22 (3.1)	
<10	211 (19.5)	129 (18.4)	
≥ 10	802 (74.1)	550 (78.5)	
**D'Amico risk stratum, N (**%**)**			0.70
Missing	298 (27.5)	165 (23.5)	
Low	241 (22.3)	171 (24.4)	
Intermediate	283 (26.2)	199 (28.4)	
High	260 (24)	166 (23.7)	
**Race, N (**%**)**			0.35
Missing	20 (1.8)	8 (1.1)	
African American	224 (20.7)	163 (23.3)	
Caucasian	790 (73)	494 (70.5)	
Others	48 (4.5)	36 (5.1)	
**PSA at CaP Diagnosis, N (**%**)**			0.27
Missing	222 (20.5)	126 (18)	
<10	582 (53.8)	371 (52.9)	
10-20	177 (16.4)	139 (19.8)	
>20	101 (9.3)	65 (9.3)	
**Pathologic Gleason Sum, N (**%**)**			0.17
Missing	227 (21)	98 (14)	
≤6	328 (30.3)	234 (33.3)	
3+4	249 (23)	201 (28.7)	
4+3	112 (10.4)	73 (10.4)	
≥ 8	166 (15.3)	95 (13.6)	
**Pathological T stage, N (**%**)**			**0.011**
Missing	54 (5)	15 (2.1)	
pT2	436 (40.3)	334 (47.6)	
pT3-T4	592 (54.7)	352 (50.3)	
**Positive Surgical Margin Status, N (**%**)**	506 (46.8)	311 (44.4)	0.16
**Positive Extracapsular Extension, N (**%**)**	511 (47.2)	265 (37.8)	**<.0001**
**Positive Seminal Vesicle Invasion, N (**%**)**	163 (15.1)	96 (13.7)	0.27

^1^CaP = prostate cancer.

^2^SD= standard deviation.

^3^RP = radical prostatectomy.

## Data Availability

For IRB protocol, the authors are not permitted to share raw data files. However, they can provide any collapsed data and statistical information that are requested.
